# Comparative effects of five polymethoxyflavones purified from *Citrus tangerina* on inflammation and cancer

**DOI:** 10.3389/fnut.2022.963662

**Published:** 2022-09-08

**Authors:** Qiyang Chen, Yue Gu, Chun Tan, Balasubramani Sundararajan, Zhenqing Li, Dan Wang, Zhiqin Zhou

**Affiliations:** ^1^School of Life Science and Engineering, Southwest University of Science and Technology, Mianyang, China; ^2^Key Laboratory of Horticulture Science for Southern Mountainous Regions of Ministry of Education, College of Horticulture and Landscape Architecture, Southwest University, Chongqing, China; ^3^The Southwest Institute of Fruits Nutrition, Chongqing, China

**Keywords:** *Citrus tangerina* “Dahongpao”, polymethoxylated flavone, tangeretin, anti-inflammation, anti-cancer

## Abstract

Although the *Citrus tangerina* cultivar “Dahongpao” (CTD) has been established as a rich source of polymethoxyflavones (PMFs) with anti-inflammatory and anti-cancer properties, their individual effects on cellular signaling remain to be elucidated. In this study, five major PMFs from the peel of CTD were isolated, including sinensetin, tetramethyl-O-scutellarin (5,6,7,4′-tetramethoxyflavone), nobiletin (5,6,7,8,3′, 4′-hexamethoxyflavone), tangeretin (5,6,7,8,4′-pentamethoxyflavone), and 5-demethylnobiletin (5-OH-6,7,8,3′,4′-pentamethoxyflavone). These PMFs were found to significantly (*p* < 0.05) inhibit the production of NO and biomarkers of chronic inflammation (TNF-α and IL-6). Additionally, they effectively suppressed mRNA biomarkers of acute inflammation (*Cox-2* and *iNOS*), and to varying degrees promoted the activation of anti-inflammatory cytokines (IL-4, IL-13, TNF-β, and IL-10). Among the five PMFs, tangeretin was found to have a considerable anti-proliferative effect on tumor cell lines (PC-3 and DU145) and synergistically enhanced the cytotoxicity of mitoxantrone, partially *via* activation of the PTEN/AKT pathway. The findings of this study provide valuable insights into the activity of different PMF monomers and advance the understanding of the roles of PMFs in promoting apoptotic and anti-cancer effects.

## Introduction

*Citrus tangerina* “Dahongpao” (CTD), a member of the Rutaceae family, is a tangerine cultivar widely grown in southwestern China. For centuries, the dried peels of mandarin/tangerine (*C. reticulata* L.), also referred to as “chenpi,” have been used to relieve and treat the symptoms of chronic or acute inflammation associated with digestive disorders (1), and recent pharmacological studies have shown that numerous components contained in aged citrus peel, particularly polymethoxylated flavones (PMFs), are the main bioactive constituents (2). PMFs are a subgroup of plant flavonoids that have attracted increasing attention on account of their broad-spectrum biological activities, including anti-cancer, anti-inflammatory, neuro-protective, anti-microbial, and anti-oxidative properties (3–5). With respect to these aforementioned properties of PMFs, the number and positions of methoxy groups have been identified as important factors contributing to their biological activity, with those containing a larger number of methoxy groups, being characterized by higher hydrophobicity, and thus stronger bioactivity (6). Although advanced phytochemical isolation and structural identification techniques have revealed more than 80 citrus PMFs (7), studies conducted to date have tended to focus on just two of these, nobiletin and tangeretin, whereas relatively little information is available regarding other PMFs (5, 7). Various PMFs showed differential effects on anti-inflammatory and anti-cancer, making it difficult to identify which activity was responsible for their protective effects.

In most acute or chronic diseases, inflammation always occurs at the beginning or end of diseases (8). Moderate inflammatory cytokines can help the body clear the invading pathogens, however, when homeostasis is disrupted, inflammation will develop into long-term diseases or acute injuries (9). Acute inflammations such as sepsis, endotoxemia, and some acute tissue damage release large amounts of pro-inflammatory cytokines and eventually break host immune tolerance (10). In response to acute infection or tissue damage, macrophages (M1 macrophages) initiate or increase the production of broad-spectrum pro-inflammatory cytokines (IL-1β, interleukin 1 beta; Cox-2, cyclooxygenase 2) and other inflammatory mediators (iNOS, inducible NO synthase) (11). Besides, tumor necrosis factor α (TNF-α) produced by macrophages also functions as a mediator of acute inflammation, platelet activation, and participation in the genesis of fever and anemia (12). Meanwhile, M2 macrophages, which are induced by anti-inflammatory cytokines including IL-4, IL-10, IL-13, and transforming growth factor β (TGF-β), are associated with anti-inflammation, wound healing, and tissue repair. Systemic chronic inflammation is a low-level, non-infectious chronic inflammatory state, which is closely related to cardiovascular disease, obesity, type 2 diabetes, various cancers, and neurodegenerative diseases. Monocytes are the precursors of the lipid-laden foam cells within atherosclerotic plaque and adipose tissue that produce sustainable levels of chronic pro-inflammatory cytokines such as TNF-α and IL-6 (13). Hence, attempts to identify anti-inflammatory agents and dietary regimens to prevent pro-inflammatory processes in the early stages of the expression of key pro-inflammatory mediators and cytokine genes have attracted a lot of interest.

Cancer has been a leading cause of morbidity and mortality worldwide for decades. Citrus PMF extracts have been used clinically to treat different types of cancer and exert anti-cancer activity in every stage. *Citrus grandis* Osbeck leaf extract, containing isosinensetin, sinensetin, tetramethyl-O-isoscutellarein, nobiletin, tangeretin and 5-demethylnobiletin, significantly reduced the survival rate of HeLa cells (14). Three major 5-hydroxy PMFs, namely: 5-demethylnobiletin, 5-hydroxy-3,6,7,8,3′,4′-hexamethoxyflavone, 5-hydroxy-6,7,8,4′-tetramethoxy-flavone, had overt inhibitory effects on human colon cancer HCT116 and HT29 cells (15). In addition, oral administration of 5-demethylnobiletin strongly lowered colon tumor incidence and reduced overall tumor size by decreasing the inflammatory mediators IL-1, IL-6, iNOS, and COX-2 in colonic tissue (16). Tangeretin inhibits cancer cells that lack the ability to repair DNA double-strand breaks (17). In addition, nobiletin arrested the cell cycle and reduced the cell viability of different cell lines. In terms of anti-cancer properties, PMFs can promote both intrinsic and extrinsic apoptotic pathways *via* the induction of p53 expression or the Bax: Bcl-2 protein ratio (7, 18), whereas different PMFs have been shown to have differential anti-proliferative activities against human cancer cell lines and circulating levels of NO (19). In addition, tangeretin can modulate energy production, inhibit cancer growth, induce apoptosis, and suppress cell cycle progression and migration in cancer cells (20). Moreover, it has recently been demonstrated that PMF monomers, including nobiletin, tangeretin, and 5-demethylnobiletin, can inhibit the proliferation of three gastric cancer cell lines and induce apoptosis (4). Interestingly, the findings of previous studies have identified a synergistic effect between certain anti-proliferative agents and tangeretin in enhancing anti-cancer activity (21). Prostate cancer is among the most prevalent malignancies and the second most prominent cause of cancer-related deaths in men (22, 23). Whereas, neuroendocrine prostate cancer accounts for ~2% of cases, it has been established that cells expressing neuroendocrine biomarkers can be detected in between 10 and 100% of prostate adenocarcinomas (24). However, although the bioactivities analyses of PMFs in different *Citrus* species have been reported (7, 19), the therapeutic efficacy of PMF monomers in prostate cancer remains poorly understood.

In this study, a macrophage-based system was used to compare the bioactivities of five major PMFs isolated from the peel of CTD on acute and chronic inflammation and anti-inflammatory factors. Moreover, the anti-proliferative properties of these compounds against human prostate cancer cell lines (PC-3 and DU145) were examined using a 3-(4,5-dimethylthiazol-2-yl)-2,5-diphenyltetrazolium bromide (MTT) assay, and the synergistic effects of co-administered mitoxantrone and tangeretin were also evaluated on these cancer cell lines.

## Materials and methods

### Standards and chemicals

Tangeretin, 3,5,6,7,8,3′,4′-heptamethoxyflavone, and 5-hydroxy-6,7,8,3′,4′-pentamethoxyflavone (5-demethylnobiletin) were obtained from ChromaDex (Santa Ana, CA, USA); isosinensetin, sinensetin, 5,6,7,4′-tetramethoxyflavone (tetramethyl-O-scutellarin), and nobiletin were obtained from SinoStandards (Chengdu, China); and HPLC-grade methanol, formic acid, dimethyl sulfoxide (DMSO), and MTT were obtained from Sigma (St. Louis, MO, USA). A Milli-Q system (Millipore, Bedford, MA, USA) was used to produce deionized water (18.20 M cm). All other analytical grade reagents were purchased from Chuandong Chemical Reagent Co., Ltd. (Chongqing, China).

### Plant materials

CTD fruits were harvested during the commercial maturity stage (in October) from the National Citrus Germplasm Repository (Chongqing, China), and authenticated by Professor Dong Jiang (Citrus Research Institute, Southwest University). A voucher specimen (voucher No. LR0094) has been deposited in Citrus Research Institute, Southwest University, Chongqing, China. The clean peel of these fruits was dried at 40°C in a circulating drying cabinet, and then ground and passed through a 60-mesh sieve.

### Extraction, enrichment, and isolation of PMFs

PMFs were prepared using the procedure described in the previous study, with minor modifications (25). Briefly, the air-dried citrus peel powder (5.0 kg) was ultrasound (300 W)-extracted twice with 100 L of 90% ethanol (each for 34 min) at 41°C and concentrated in vacuo to obtain a crude extract. This extract was diluted in 25% ethanol prior to loading onto an HPD 300 macroporous resin-based column chromatography system. Following adsorption, the impurities were removed by eluting with distilled water and 7% ethanol. A PMF-rich extract was obtained by subsequent elution with 90% ethanol, and following rotary evaporation to remove all organic reagents, was frozen at −80°C and freeze-dried to obtain a lyophilized powder. The freeze-dried PMF-rich extract (5.15 g) was subsequently redissolved in methanol to a concentration of 100 mg/mL, from which individual PMFs were separated and isolated using a prep-HPLC system equipped with a 515 HPLC Pump, a Waters 2767 Sample Manager, an ACQUITY QDa detector, and an XBridge Shield RP18 column (19 × 250 mm, 5 μm; Milford, MA, USA).

### PMF identification

To identify the constituents of the PMF-rich extract and determine the purity of individual PMFs, we performed ultra-high-performance liquid chromatography-photodiode array (UPLC-PDA) spectrometry (Waters, Milford, MA, USA). Samples were separated on an ACQUITY UPLC BEH C18 column (2.1 × 100 mm, 1.7 mm, UK) at a column temperature of 50°C. The mobile phases were water containing 0.05% formic acid (solvent A) and methanol (solvent B), with gradient elution performed as follows: 0–0.6 min, 90–80% A; 0.6–5 min, 80–30% A; 5–7 min, 30–10% A; 7–9 min, 10–90% A. The flow rate was set to 0.3 mL/min and the detection wavelength was 330 nm. Nuclear magnetic resonance (NMR) spectra were obtained using a Bruker NMR AVANCE III10600 spectrometer (Bruker BioS-pin, Karlsruhe, Germany).

### Cell culture

Murine macrophage RAW264.7 cells (ATCC®, Chicago, USA) were incubated in Dulbecco's Modified Eagle's Medium (DMEM; Fisher) containing 10% (v/v) fetal bovine serum (FBS; BI, Israel) and 1% (v/v) penicillin-streptomycin (Gibco™, USA). The human prostate cancer cell lines PC-3 and DU145 (ATCC®, Chicago, USA) were maintained in 50% DMEM and 50% F-12K medium (Fisher) supplemented with 10% FBS and 1% penicillin-streptomycin. These cell lines were incubated at 37°C in a 5% CO_2_ humidified incubator. PMF samples used for cell assays were prepared as 1,000 × stock solutions in DMSO and unless otherwise stated, were maintained at 20°C until used.

### Cell viability assay

RAW264.7 cells were seeded in 96-well plates at a density of 8 × 10^3^ cells/well and left to stabilize for 24 h. The viability of cells treated with PMFs was determined after a 48-h incubation using an MTT assay, in which sextuplicate samples were quantified spectrophotometrically at 570 nm using a Synergy H1 microplate reader (BioTek, Winooski, Vt., USA). The concentrations of test reagents that promoted no changes in the viability of RAW264.7 cells compared with that of the vehicle were selected for further studies.

### Anti-inflammatory activity assay

RAW264.7 cells (5 × 10^6^ cells/3 mL/dish in 35-mm culture dishes) were pretreated with PMFs (25 μM) or DMSO for 24 h, after which the cells were treated with fresh medium containing 1 μg/mL LPS and incubated for a further 24 h. Dexamethasone (DEX, 10 μM) was employed as a positive control. NO production in the supernatant was determined using a Griess reagent system (Beyotime, Beijing, China) according to the manufacturer's instructions. Absorbance was determined at 540 nm. The levels of anti-inflammatory cytokines (IL-4, IL-10, IL-13, and TGF-β) and pro-inflammatory cytokines (IL-6, IL-1β, and TNF-α) in the supernatant were measured using enzyme-linked immunosorbent assay kits (Shanghai Optimization Biological Technology Co., LTD.) according to manufacturer's protocol.

### Anti-proliferative activity assay

For the purposes of anti-proliferative activity assays, following cell stabilization, the medium was replaced with fresh media containing differing amounts of test compounds (6.25, 12.5, 25, 50, 100, and 150 μM), and the cell viability was determined after incubating for a further 48 h, as described in Section Cell viability assay. Anti-proliferative activity was determined using the following equation:


$$\begin{array}{l} \text{Anti}—\text{ proliferative activity}(\%)\text{ = }(\text{1}-(\text{OD experiment})/\\ \;(\text{OD control})\times \text{100} \end{array}$$

### Morphological assay

Clonogenic assays were performed using cells (1 × 10^3^ cells/well) seeded in 6-well plates and pretreated with tangeretin (12.5 μM), mitoxantrone (10 nM), or the combination thereof for 24 h. Thereafter, the reagent-containing medium was replaced with fresh medium and the treated cells were incubated for a further 7 days. The resulting colonies were stained with 0.25% crystal violet for counting.

For nuclear morphology assays, cells (1 × 10^4^ cells/well) were seeded in 24-well plates and left for 24 h. Thereafter, the stabilized cells were treated with tangeretin (12.5 μM), mitoxantrone (10 nM), or the combination thereof for 48 h. The cells were then fixed with 4% paraformaldehyde for 30 min and stained with 1 μg/mL 4′,6′-diamidino-2-phenylindole for 15 min at room temperature. The cells were subsequently washed with phosphate-buffered saline and viewed under an Olympus BX53 upright fluorescence microscope (Olympus). Images were captured using a DP27 digital camera and cellSens Dimension software.

### Total RNA extraction, purification, and cDNA synthesis

Cells were harvested in RNase-free EP tubes, from which total RNA was extracted using TRIzol reagent (Invitrogen). cDNA synthesis using a reverse transcription cDNA synthesis kit (TIANGEN Biotech, Beijing) and quantitative PCR were performed using a Step One Plus Real-Time PCR Systems under the following PCR cycling conditions: 95°C for 1 min; 95°C for 20 s (45 cycles), and 60°C for 60 s. The oligonucleotide primers used for amplification (Supplementary Table 1) were synthesized by Beijing Hua-da Genetic Engineering Company. β-actin was used as an internal reference gene, and relative gene expression was calculated using the 2^−Δ*ΔCt*^ method (26).

### Western blotting

PC-3 cells (5 × 10^6^ cells/well) were seeded in 80-mm dishes, left to stabilize for 24 h, and then treated with tangeretin, mitoxantrone, or the combination thereof for a further 48 h. Cytoplasmic proteins were extracted using the procedure described by Yoon and Liu (27) and a BCA kit (Beyotime, China) was used to determine protein contents. Equal amounts of isolated protein samples were separated using 10% SDS-PAGE gels, and the separated proteins were subsequently transferred to PVDF membranes (Merck Millipore). The membranes were then blocked for 1 h at room temperature with non-fat dry milk (5%) in tris buffer containing Tween-20 solution (TBST, 0.1%), prior to being probed with primary antibodies (Bcl-XL, Caspase-9, and Caspase-3) overnight at 4°C. The membranes were then incubated for 1 h at room temperature in TBST solution containing a horseradish peroxidase-conjugated second antibody. Protein bands were visualized using Odyssey CLX infrared imaging system (LICOR Bioscience).

### Data analysis

Data are presented as the means ± standard deviation (SD). The data were analyzed by one-way analysis of variance (ANOVA) using the statistical package for social sciences software (SPSS 22.0; IBM, USA). A *p*-value < 0.05 was taken to be indicative of statistical significance.

## Results and Discussion

### Quantitative analysis and isolation of PMFs

**Figure 1:**
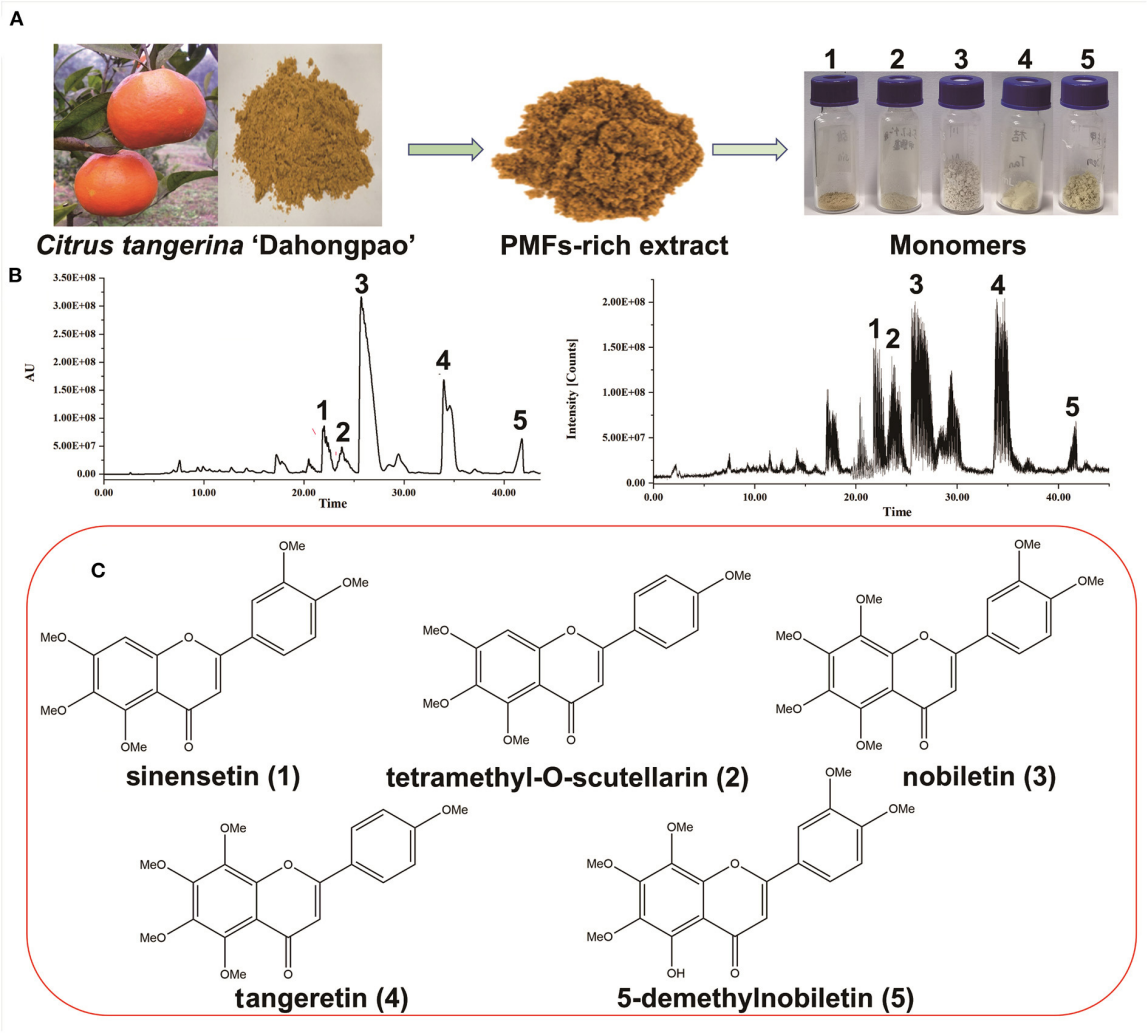
Purification and identification of polymethoxyflavones (PMFs) from *Citrus reticulata* “Dahongpao.” **(A)** The preparation of PMFs; **(B)** Chromatogram and MS^E^ ion profile of PMFs; **(C)** Chemical structure of PMFs.

The seven main PMF compounds, namely six OCH_3_-PMFs and one OH-PMF, were quantitatively detected in the peel extract of “Dahongpao” tangerine by UPLC–PAD (Supplementary Figure 1). The PMF-rich extract from CTD contained nobiletin (210.87 ± 0.57 mg/g extract), tangeretin (55.66 ± 0.62 mg/g), sinensetin (28.11 ± 1.11 mg/g), 5-demethylnobiletin (24.66 ± 0.33 mg/g), isosinensetin (12.90 ± 0.12 mg/g), tetramethyl-O-scutellarin (12.14 ± 0.36 mg/g), and 3,5,6,7,8,3′,4′- heptamethoxyflavone (7.68 ± 0.46 mg/g) as the major PMF components (Supplementary Table 2). Consistent with the findings of previous studies (25), nobiletin and tangeretin were the PMFs detected in the highest amounts in the tangerine peel. To further evaluate the activity of these metabolites, five PMFs from the peel of CTD were separated and purified using MS-directed prep-HPLC (Figure 1). The yields and spectroscopic data of these compounds are shown in Table 1 and Supplementary Figures S2–S11.

**Table 1:** The yield and spectroscopic data for isolated citrus PMFs.

**Compound**	**Yield (mg)**	**[M+H]^+^ (error, mDa)**	**MS^E^ ion fragments (%)**	**^1^H NMR (400 MHz, CDCL_3_): δ (ppm)**	**^13^C NMR (400 MHz, CDCL3): δ (ppm)**
Sinensetin	127.82 (purity > 99%)	373.1280 (−0.1)	395.11060 (100.00) 343.08177 (42.74) 312.09092 (6.78) 359.07851 (20.91) 329.10161 (16.23)	7.58–7.44 (m, 1H), 7.28 (s, 0H), 6.97 (d, *J* = 8.5 Hz, 1H), 6.81 (s, 1H), 6.62 (s, 1H), 4.00 (s, 6H), 3.98 (s, 2H), 3.96 (s, 3H), 3.93 (s, 3H)	177.25 (C-4), 161.18 (C-2), 157.69 (C-7), 154.49 (C-5), 152.57 (C-9), 151.83 (C-4'), 149.28 (C-3'), 140.37 (C-6), 124.09 (C-1'), 119.60 (C-6'), 112.83 (C-10), 111.15 (C-5'), 108.71 (C-2'), 107.33 (C-3), 96.25 (C-8), 62.17 (OMe), 61.52 (OMe), 56.31 (OMe), 56.13 (OMe), 56.07 (OMe)
Tetramethyl-O-scutellarin	90.52 (purity > 95%)	343.1173 (0.1)	365.10020 (100.00) 313.07135 (98.42) 343.11732 (28.73) 285.07609 (28.63) 314.07457 (27.29)	7.90 (d, *J* = 2.2 Hz, 1H), 7.88 (d, *J* = 2.0 Hz, 1H), 7.03 (d, *J* = 2.2 Hz, 1H), 7.02 (d, *J* = 2.1 Hz, 1H), 6.61 (s, 1H), 6.44 (s, 1H), 4.01 (s, 4H), 3.99 (d, *J* = 2.6 Hz, 3H), 3.96 (s, 3H), 3.89 (s, 3H)	177.84 (C-4), 162.21 (C-2), 160.70(C-4'), 156.46 (C-5),156.33 (C-5), 127.72 (C-2'), 123.96 (C-1'), 114.49 (C-5'), 109.18 (C-10), 107.04 (C-3), 92.75 (C-8), 61.58 (OMe), 56.65 (OMe), 56.33 (OMe), 55.49 (OMe)
Nobiletin	1280.46 (purity > 99%)	403.1380 (−0.1)	373.09604 (78.67) 374.09550 (33.13) 395.07456 (27.89) 388.11481 (22.68) 355.08157 (12.16)	7.63–7.58 (m, 1H), 7.58–7.54 (m, 1H), 7.44–7.40 (m, 3H), 7.27 (d, *J* = 1.0 Hz, 2H), 7.01 (d, *J* = 1.0 Hz, 1H), 6.99 (d, *J* = 1.0 Hz, 1H), 6.64 (d, *J* = 1.1 Hz, 2H), 5.47 (s, 0H), 4.11 (d, *J* = 1.1 Hz, 6H), 4.03 (d, *J* = 1.1 Hz, 7H), 3.98 (d, *J* = 1.1 Hz, 7H), 3.97 (d, *J* = 1.1 Hz, 7H), 3.96 (d, *J* = 1.1 Hz, 11H)	177.31 (C-4), 161.08 (C-2), 152.04 (C-4'), 151.46 (C-7), 149.42 (C-3'), 148.47 (C-5), 147.74 (C-9), 144.15 (C-6), 138.07 (C-8), 124.13 (C-1'), 119.67 (C-6'), 114.92 (C-10), 111.37 (C-5'), 108.77 (C-2'), 106.93 (C-3), 62.27 (OMe), 61.95 (OMe), 61.82 (OMe), 61.66 (OMe), 56.12 (OMe), 56.04 (OMe)
Tangeretin	526.41 (purity > 99%)	373.1280 (−0.1)	395.11048 (100.00) 343.08166 (89.54) 344.08493 (23.10) 358.10504 (13.27) 328.05830 (8.84)	7.93–7.84 (m, 2H), 7.07–6.99 (m, 2H), 6.62 (s, 1H), 4.11 (s, 3H), 4.02 (s, 3H), 3.95 (*J* = 0.7 Hz, 6H), 3.89 (s, 3H)	177.30 (C-4), 162.34 (C-2), 161.21 (C-4'), 151.40 (C-7), 148.44 (C-5), 147.76 (C-9), 144.13 (C-6), 138.14 (C-8), 127.74 (C-2',6'), 123.95 (C-1'), 114.95 (C-10), 114.56 (C-3',4'), 106.76 (C-3), 62.26 (OMe), 62.02 (OMe), 61.82 (OMe), 61.64 (OMe), 55.51 (OMe)
5-demethylnobiletin	122.74 (purity > 98%)	389.1226 (−0.5)	359.07641 (100.00) 411.10530 (84.93) 341.06203 (38.80) 360.07992 (18.99) 169.00841 (5.29)	12.55 (s, 1H), 7.61 (d, *J* = 2.1 Hz, 1H), 7.58 (d, *J* = 2.1 Hz, 1H), 7.43 (d, *J* = 2.1 Hz, 1H), 7.02 (s, 1H), 7.00 (s, 0H), 6.62 (s, 1H), 4.12 (s, 3H), 3.99 (s, 3H), 3.98 (s, 4H), 3.98 (s, 3H), 3.96 (s, 3H)	183.00 (C-4), 163.96 (C-2), 153.03 (C-7), 152.58 (C-4'), 149.60 (C-3'), 149.51 (C-9), 136.68 (C-6), 133.03 (C-8), 123.82 (C-1'), 120.19 (C-6'), 111.41 (C-5'), 108.97 (C-2'), 107.06 (C-10), 104.06 (C-3), 62.05 (OMe), 61.72 (OMe), 61.13 (OMe), 56.16 (OMe), 56.07 (OMe)

### Effect of different citrus PMFs on the viability of RAW 264.7 cells

**Figure 2:**
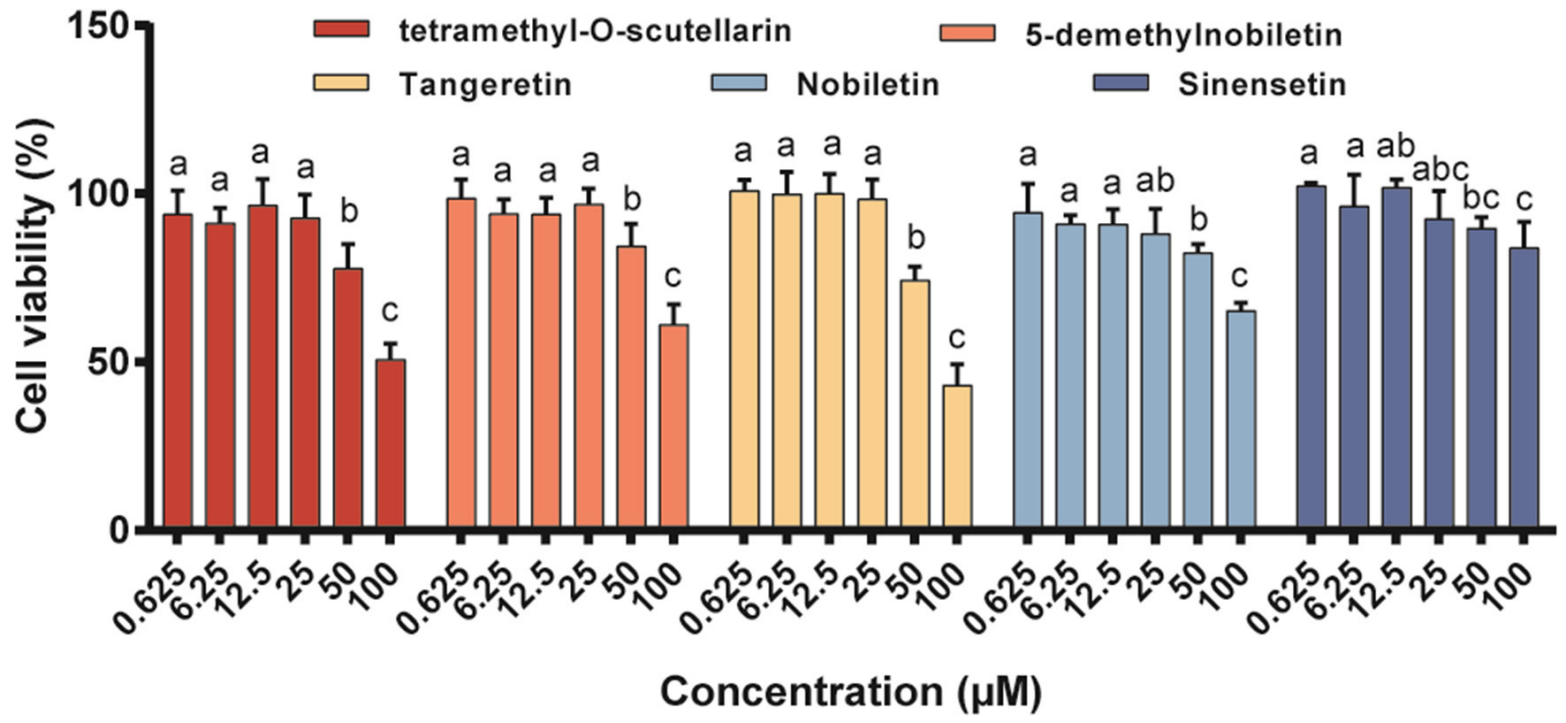
Effects of different PMFs on cell viability of murine RAW 264.7 macrophages. Data were presented as the means ± SD for three independent experiments. Means denoted by different lower-case letters were significantly different (One-way ANOVA, Dunnett's *post-hoc* test, *p* < 0.05).

The cytotoxic effects of the five isolated PMFs on RAW 264.7 macrophage viability after 48 h of exposure were assessed using the MTT assay (Figure 2). The results revealed that none of these PMFs had any adverse effects regarding cell viability in the concentration range 0.625–25 μM, which we accordingly used in the subsequent studies. However, when applied at 50 μM, all five PMFs significantly reduced cell viability to levels below 90% and the inhibitory effects were enhanced at higher concentrations.

### Effect of different citrus PMFs on inflammatory biomarkers

**Figure 3:**
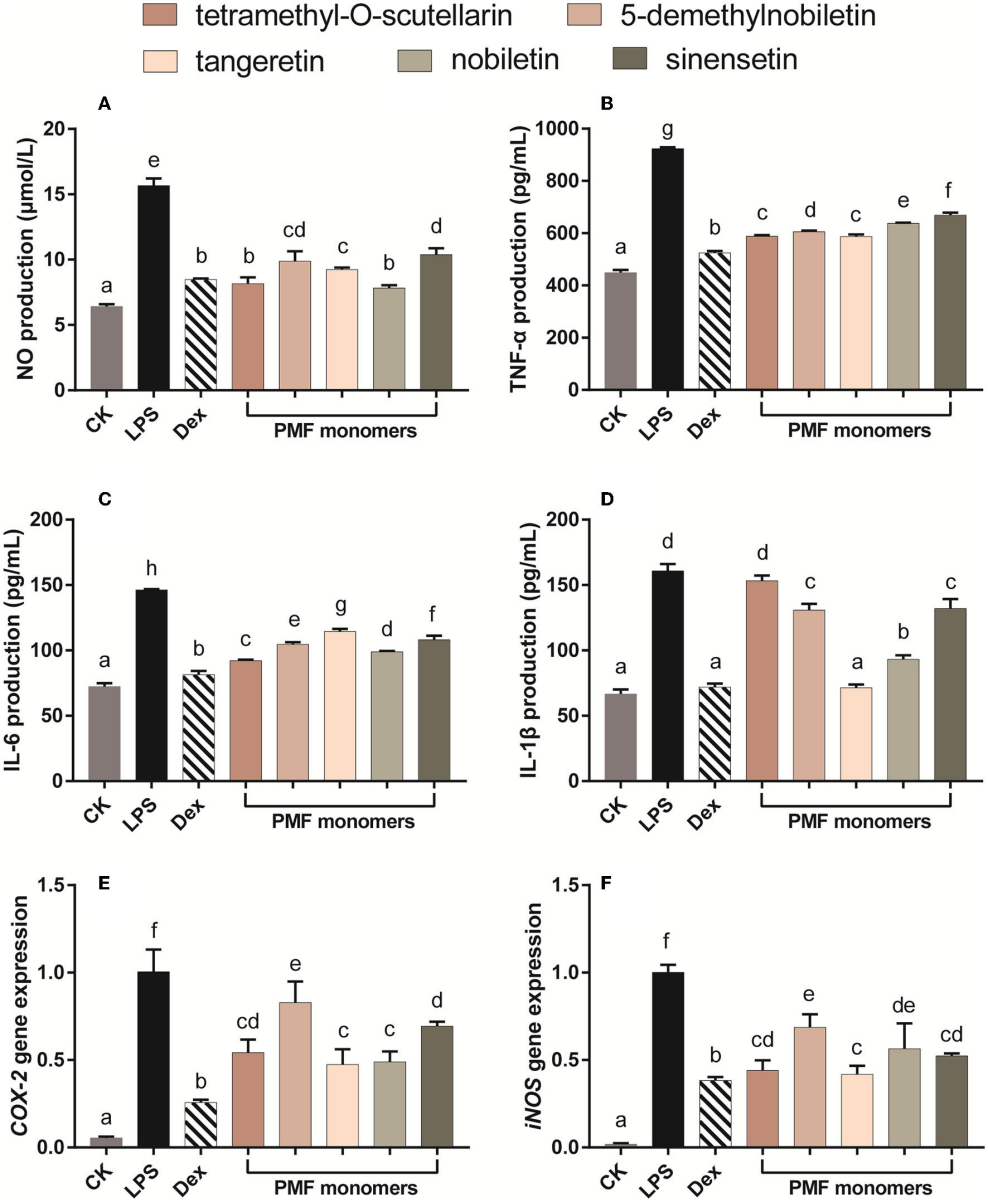
Effects of different PMFs on NO production **(A)**, pro-inflammatory cytokine and gene expression profiles associated with chronic inflammatory **(B,C)** and acute inflammatory response **(D–F)** in the LPS-stimulated RAW264.7 macrophage cells. Results are expressed as means ± SD, *n* = 3 experiments. Means not sharing the same letter were significantly different (One-way ANOVA, Dunnett's *post-hoc* test, *p* < 0.05 was considered significant).

The utility of LPS as a systemic inducer of inflammatory responses is based on its dual effects of inducing low-level chronic inflammation associated with gastrointestinal dysfunction and increased endotoxin flux (28), as well as endothelial cell damage and an acute inflammatory response (29). In the present study, individual PMFs were incubated with LPS-activated Raw 264.7 macrophages to ascertain cellular inflammatory activity. In this regard, nitric oxide (NO) is a highly reactive biological messenger that is overexpressed in inflammatory reactions and different diseases and can induce DNA damage (30), The control of intracellular NO production accordingly represents an effective approach for inhibiting inflammatory responses (19). When LPS was administrated alone, macrophages showed an approximately 2.5-fold increase in NO production compared with that observed in control cells (Figure 3A). In contrast, all assessed PMFs significantly inhibited the production of NO. Notably, when applied at a concentration of 25 μM, tetramethyl-O-scutellarin and nobiletin inhibited NO induction to the same level as the positive control (10 μM dexamethasone).

With the exception of the inhibitory effect of tetramethyl-O-scutellarin on IL-β, all assessed PMF monomers induced a significant suppression of all evaluated biomarkers of inflammation (Figures 3B–F). With respect to TNF-α production, the inhibitory capacity of the five PMFs could be ordered as follows: tetramethyl-O-scutellarin = tangeretin > 5-demethylnobiletin > nobiletin > sinensetin. Similarly, in the case of IL-6 production, the inhibitory effects could be ordered as follows: tetramethyl-O-scutellarin > nobiletin > 5-demethylnobiletin > sinensetin > tangeretin. These findings are comparable to those previously reported for three polymethoxyflavones (nobiletin, tangeretin, and 5-demethylnobiletin) isolated from ougan (*C. reticulata* Cv. Suavissima), which have been found to inhibit NO production, and the expressions of IL-1β, IL-6, and TNFα (31). Among these targets, IL-1β expression was found to be the most strongly affected by treatments, with the exception of tetramethyl-O-scutellarin, which showed no significant effect regarding the attenuation of LPS-induced IL-1β expression. Notably, tangeretin was found to be the only monomer showing borderline inhibition of IL-β at 25 μM (*p* < 0.05). In addition, the expression of the pro-inflammatory genes *Cox-2* and *iNOS* was found to be strongly suppressed by all monomers, with tangeretin being the most effective in this regard. Nobiletin significantly inhibited LPS-induced PGE 2 production and IL-1α, IL-1β, TNF-α, and IL-6 in mouse J774A.1 macrophages. Especially, nobiletin selectively down-regulated the expression level of *COX-2* instead of *COX-1*(32). The administration of tangeretin strongly alleviated the inflammatory injury in the kidney triggered by cisplatin in rats possibly through decreasing TNF-α level and augmenting IL-1α in molecular level (33). Moreover, oral administration of nobiletin effectively suppressed tumor formation and metastasis in the xenograft mice, and lowered the levels of NF-κB in the isolated tumors (34). It has been established that the anti-inflammatory activity of citrus flavonoids is closely associated with the structural properties of different flavonoids (35), and in this respect, it is speculated that the strong inhibitory capacities of tangeretin and nobiletin against IL-β and *Cox-2* are correlated with the methoxy substituents at positions 5 and 8 (36). Consistent with the inhibitory effect of NO production, we speculate that the hydroxyl group may be associated with attenuating the inhibitory activity of PMFs on the expression of *iNOS* (36). In summary, these findings indicate that differences in the inhibitory effects of PMFs against biomarkers of inflammation could be attributable to difference their molecular structures (37).

### Effect of different citrus PMFs on anti-inflammatory cytokines

**Figure 4:**
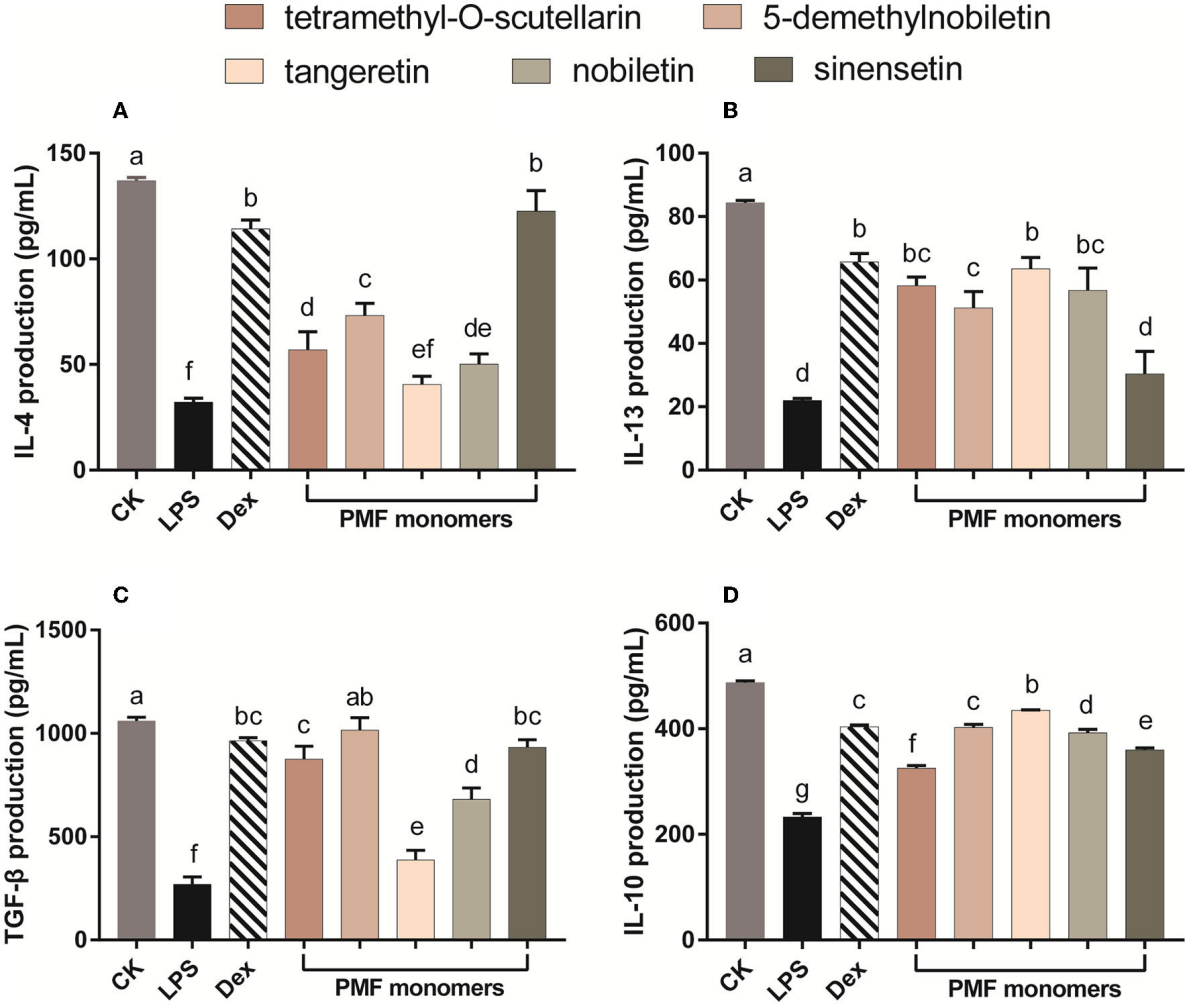
Effect of different PMFs on anti-inflammatory cytokines in the LPS-stimulated RAW264.7 macrophage cells. **(A)** IL-4; **(B)** IL-13; **(C)** TGF-â; **(D)** IL-10. Results were expressed as means ± SD, *n* = 3 experiments. Means denoted by different lower-case letters are significantly different (One-way ANOVA, Dunnett's *post-hoc* test, *p* < 0.05).

Cytokines, which include both pro-inflammatory and anti-inflammatory types, are well-established regulators of host responses to infection, immune responses, inflammation, and trauma. Inflammatory processes are controlled by anti-inflammatory cytokines, including IL-10 and IL-4, the activity of which contributes to the inhibition of the production of pro-inflammatory cytokines such as IL-1, IL-6, and TNF-α (38). Exposure of murine RAW 264.7 macrophages cells to LPS leads to excessive production of pro-inflammatory cytokines, which in turn initiates the activation of inflammatory cascades (1). In the present study, the levels of the anti-inflammatory cytokines IL-4, IL-13, TNF-β, and IL-10 in LPS-activated Raw 264.7 macrophages were increase in response to incubation with most of the assessed individual PMF (Figure 4). IL-4 production was most strongly affected by sinensetin, which showed no difference with the positive control (Figure 4A). The activation capacity for IL-13 was in the order of tangeretin ≥ tetramethyl-O-scutellarin ≥ nobiletin ≥ 5-demethylnobiletin > sinensetin (Figure 4B). TGF-β plays an essential role in the chemotaxis of macrophage and fibroblast toward wounds, and the interaction between macrophages and TGF-β has been established to contribute to wound repair (39). In the present study, 5-demethylnobiletin, sinensetin, tetramethyl-O-scutellarin, and nobiletin were generally effective in promoting an increase in TGF-β (Figure 4C). It has also been established that the phenotype of macrophages undergoes a transition from M1 to M2 in response to the up-regulated expression of IL-10 (40). We found that the individual citrus PMFs had a significant promotive effect on IL-10, with tangeretin being observed to strongest capacity in this regard (Figure 4D). On the basis of the findings with respect to IL-10, we speculated that IL-10/STAT3 signal-mediated macrophage phenotypic transition is one of the pathways *via* which PMFs contribute to an alleviation of LPS-induced inflammation (40). In summary, on the basis of comparative analysis of the structures of the five assessed PMF monomers, the difference in the mechanisms of action could be associated with the number and position of methoxy groups and the presence of hydroxyl groups.

### Effect of different citrus PMFs on anti-proliferative activity

**Figure 5:**
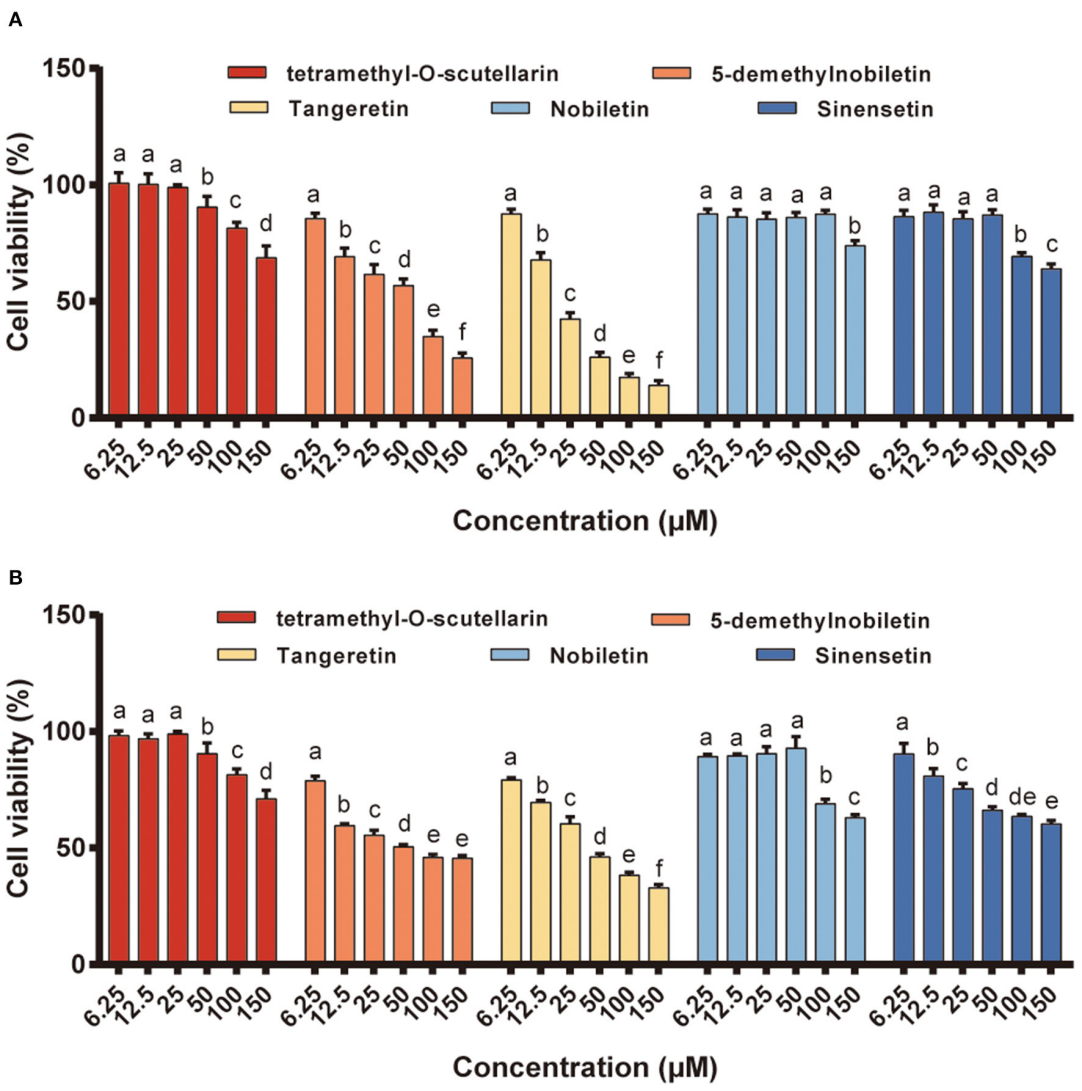
The anti-proliferation activity of different PMFs against two prostate cancer cell lines. **(A)** PC-3; **(B)** DU145. Results were expressed as the means ± SD, *n* = 3 experiments. Means denoted by different lower-case letters are significantly different (One-way ANOVA, Dunnett's *post-hoc* test, *p* < 0.05).

Compared with that in normal cells, reactive oxygen species (ROS) homeostasis in cancer cells is maintained at a generally higher level and that the prostate cancer cells PC-3 and DU145 are characterized by higher ROS generation than normal prostate cells (41, 42). In addition, up to 25% of the lethal metastatic cancers have been shown to comprise neuroendocrine cells, and the PC-3 cell line is characterized by a neuroendocrine or small cell carcinoma type rather than an adenocarcinoma type (43, 44). In the present study, two different human prostate cancer cell lines (DU145 and PC-3) were used to evaluate the anti-proliferative activity of the different citrus PMFs (Figure 5), the IC_50_ values (concentration of the test compound that causes 50% inhibition) of which are listed in Supplementary Table 3. Among the assessed PMFs, tangeretin and 5-demethylnobiletin exhibited significant anti-proliferative activity against both these cell lines, whereas no appreciable inhibitory effects among cells treated with the other three PMFs (tetramethyl-O-scutellarin, nobiletin, and sinensetin) were observed at the assessed concentrations. In this context, the findings of previous studies have indicated that hydroxylation of PMFs at the 5-position of the A-ring is an essential pre-requisite for enhancing their anti-cancer activity (45), which can probably be ascribed to the formation of ionic bonds between the free phenolic hydroxyl group and charged amino acid residues (19). Indeed, these results confirmed that 5-demethylnobiletin was characterized by a stronger anti-proliferative activity than nobiletin. However, compared with 5-demethylnobiletin, tangeretin exhibited a notably stronger cytotoxic effect against the two assessed prostate cancer cell lines, with IC_50_ values of 22.12 and 46.60 μM for PC-3 (Figure 5A) and DU145 (Figure 5B), respectively. Available studies suggest that citrus PMFs can exert anticancer activity in six aspects, including reducing tumorigenesis by absorbing environmental carcinogens or eliminating mutations *in vivo* (17); promoting apoptosis by regulating apoptotic protein expression (20); inhibiting cancer cell proliferation or blocking nutrient and oxygen entry into cancer cells during the cell cycle; inhibiting tumor cell invasion or metastasis by suppressing matrix metalloproteinase expression; antagonizing multidrug resistance by inhibiting the activity of the ATP-binding cassette family of membrane transport proteins (46). Tangeretin and 5-demethylnobiletin. have been found to selectively upregulate expression of the *RARB* gene and activate Caspase-3 and−9 and PARP1 proteins to inhibit the proliferation of three gastric cancer cell lines and induce apoptosis. In addition to 5-demethylnobiletin and tangeretin upregulate the expression of cleaved Caspase 8, thereby implying that the pathway of 5-demethylnobiletin-induced apoptosis might differ slightly from that induced by tangeretin, which could be attributable to the hydroxy substitution at position 5 in the 5-demethylnobiletin molecule (4). Moreover, it has been established that PMFs are characterized by higher lipophilicity and greater permeability than hydroxy-PMFs, thereby tending to indicate that the potent bioactivity of PMFs may enhance cellular absorption (47). Collectively, our findings thus indicate that tangeretin is an effective anti-cancer agent that may potentially serve as a novel therapeutic option for the treatment of prostate cancer.

### Tangeretin enhances the cytotoxicity of mitoxantrone in human prostate cancer DU145 and PC3 cell lines

Most malignancies are challenged by intrinsic drug resistance, and the combination of nature products with anticancer drugs is an intriguing new approach for cancer chemotherapy (7). Currently, mitoxantrone is commonly used as an anti-tumor drug for the effective treatment of different types of malignant tumor, including breast and advanced prostate cancers, lymphoma, and leukemia (48). Consistent with our findings that among the five assessed PMFs, tangeretin is the most effective anti-prostate cancer constituent in CTD, it has previously been reported that this PMF can enhance the bioavailability of anti-cancer agents by inhibiting the activity of efflux transporters (49). This accordingly prompted us to assess the therapeutic effects of combinations of anti-cancer drugs and tangeretin on prostate cancer.

**Figure 6:**
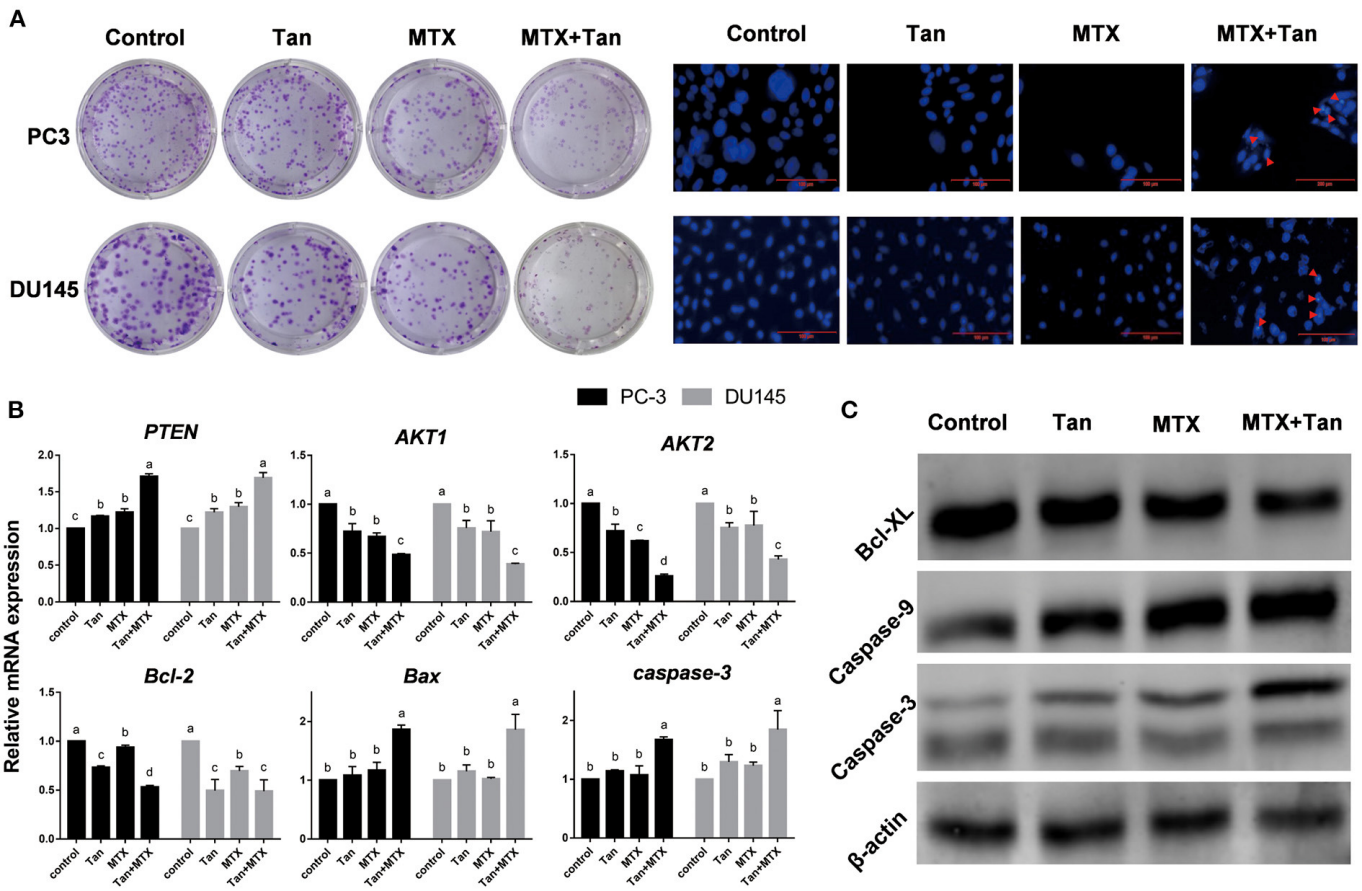
Morphology and gene and protein expression regulated by tangeretin (Tan), mitoxantrone (MTX), or their combination (MTX+Tan) in prostate cancer cell lines. **(A)** Images of clonogenic assay- and DAP-stained cells. The red arrowheads indicate apoptotic bodies. **(B)** Relative gene expression detected by qRT-PCR. **(C)** Protein expression detected by western blot analysis.

On the basis of the results of clonogenic assays, tangeretin (12.5 μM) and mitoxantrone (12.5 nM) inhibited the cell colony formation of PC3 and DU145 cells when applied alone, which is consistent with the previously reported inhibition of clonogenic survival by tangeretin or mitoxantrone (48, 50). Having established the individual activities of these two agents, co-treatments with tangeretin and mitoxantrone was performed to examine any potential synergistic inhibitory effects with respect the cell colony-forming ability of the PC3 and DU145 lines (Figure 6A). Compared with the individual treatment groups, cells exposed to a combination of tangeretin and mitoxantrone showed clear evidence of apoptotic effects, including morphological shrinkage and nuclear condensation (Figure 6A). Apoptosis is known to be primarily attributable to the caspase (cysteinyl, aspartate-specific proteases) family of proteases (51). The anti-cancer properties of tangeretin with respect to prostate cancer cells has be found to be mediated via the PI3K/Akt/mTOR signaling pathway, regulated by the *Bax, Bcl-2, Bcl-xL, caspase 9, caspase 3, PTEN, AKT*, and mTOR genes (46), and one of the hallmarks of apoptosis of apoptosis in numerous cell types is the activation of Caspase-3,−7,−8, and−9 (52). In both PC3 and DU145 cell lines, identical patterns of apoptosis-related genes expression in response to treatment with tangeretin (Figure 6B). Consistent with previously reported observations (53), the levels of *PTEN* expression in both tangeretin and mitoxantrone groups were significantly (p < 0.05) higher than those detected in control group cells, whereas in contrast, the expression of *AKT1, AKT2*, and *Bcl-2* mRNA was found to be significantly (*p* < 0.05) inhibited by either of these two compounds. However, compared with that seen in the controls, no significant changes were detected in the mRNA levels of *Bax* or *caspase-3* in cells exposed to either tangeretin or mitoxantrone. Interestingly, however, simultaneously exposing cells to both tangeretin and mitoxantrone was observed to promote significant changes in the expression of *Bax* and *caspase-3*, and western blot analysis further confirmed that when co-administered, tangeretin and mitoxantrone markedly enhanced their individual effects by suppressing the expression of Bcl-XL and upregulating the expression of Caspase-9 and−3 (Figure 6C). Accordingly, compared with their individual application, administering tangeretin and mitoxantrone in combination contributed to an enhancement of anti-cancer efficacy. Growing studies have proved that tangeretin develop as a secure and useful antagonist of multidrug resistance. For example. tangeretin has synergistic cytotoxicity with imatinib in K562 cells through an apoptotic mechanism (54). In addition, compared with doxorubicin alone, the combination of doxorubicin and tangeretin improved the cytotoxic effect on MCF-7 and T47D cells (55). The potential mechanism involves significant inhibition of the ABCB1 transporter activity and reduced efflux of ABCB1 substrates by tangeretin (56).

## Conclusion

A distinctive citrus variety (Dahongpao) with a high content of PMFs was selected as the material to separate and purify five PMF monomers (sinensetin, 5,6,7,4′-tetramethoxyflavone, nobiletin, tangeretin, and 5-demethylnobiletin). All five PMFs were found to suppress LPS-induced NO release and reduced the production of chronic inflammation-related cytokines (TNF-α and IL-6) and the expression of acute inflammatory biomarkers (*Cox-2* and *iNOS*). In addition, we demonstrated that these five compounds selectively activated different anti-inflammatory cytokines, with differential effects being ascribed to differences in molecular structure. Among the PMFs assessed, tangeretin showed the most pronounced anti-proliferative activity against prostate cancer cell lines (PC-3 and DU145) and was found to synergistically enhance the cytotoxic effects of mitoxantrone on these cells by modulating apoptosis-related genes and proteins. Collectively, our findings provide valuable information that will contribute to promoting the further utilization of CTD peel and individual PMFs as effective anti-inflammatory and anti-cancer agents.

## Data availability statement

The original contributions presented in the study are included in the article/Supplementary material, further inquiries can be directed to the corresponding authors.

## Author contributions

QC and ZZ: conceptualization. QC, CT, and YG: methodology and investigation. QC and DW: formal analysis, writing—original draft preparation, and writing—review and editing. BS and ZL: software and visualization. ZZ, QC, and DW: funding acquisition. All authors have read and agreed to the published version of the manuscript.

## Funding

This research was funded by Applied Basic Research Program of Science and Technology Department of Sichuan Province (22NSFSC2544 and 22NSFSC2571), the Ph.D. Programs Foundation of Southwest University of Science and Technology (No. 22zx7111 and 22zx7112), and National Natural Science Foundation of China (No. 31772260).

## Conflict of interest

The authors declare that the research was conducted in the absence of any commercial or financial relationships that could be construed as a potential conflict of interest.

## Publisher's note

All claims expressed in this article are solely those of the authors and do not necessarily represent those of their affiliated organizations, or those of the publisher, the editors and the reviewers. Any product that may be evaluated in this article, or claim that may be made by its manufacturer, is not guaranteed or endorsed by the publisher.
