# Supplementary material for: Comparative effects of five polymethoxyflavones purified from Citrus tangerina on inflammation and cancer
Source: Front Nutr. 2022 Sep 8;9:963662. doi: 10.3389/fnut.2022.963662 (PMC9493082; doi:10.3389/fnut.2022.963662)
Supplement: Supplementary file 1 [file Data_Sheet_1.docx]

Supplementary Material

# Supplementary Figures and Tables

## Supplementary Figures


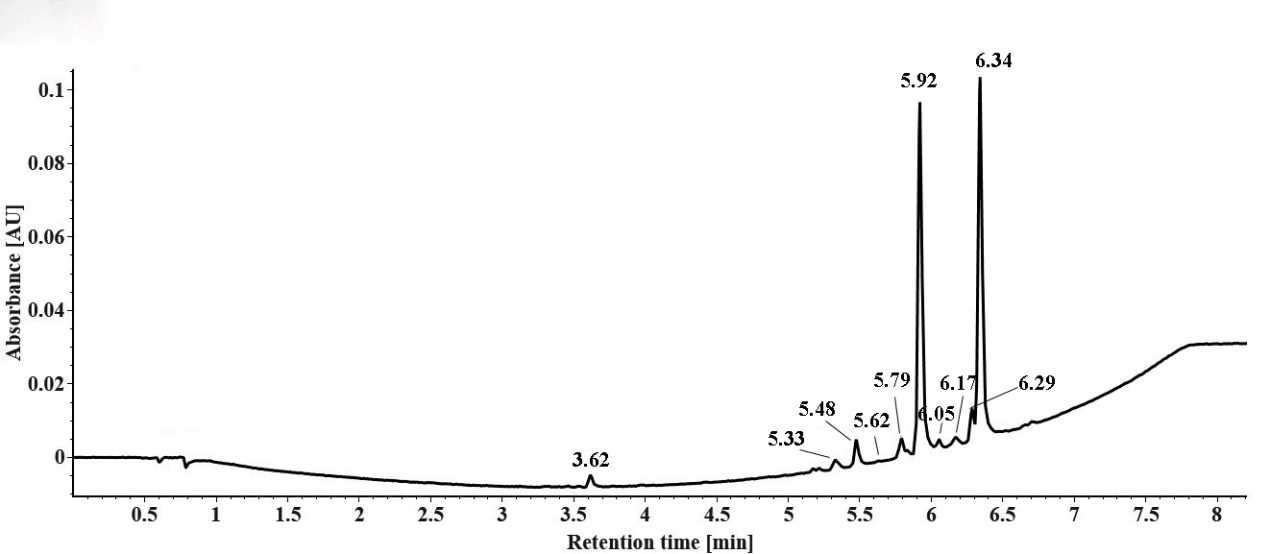


**Figure S1.** Characteristic UPLC-PDA chromatogram of *Citrus tangerina* ‘Dahongpao’ peel. 5.33 min, isosinensetin (No. 1); 5.48 min, sinensetin (No. 2) 5.79 min, 5,6,7,4′- Tetrathoxyflavone (No. 3); 5.92 min, nobiletin (No. 4); 6.06 min, 3,5,6,7,8,3′,4′- heptamethoxyflavone (No. 5); 6.29 min, 5-Hydroxy-6,7,8,3′,4′-pentamethoxyflavone (No. 6); 6.34 min, tangeretin (No. 7).


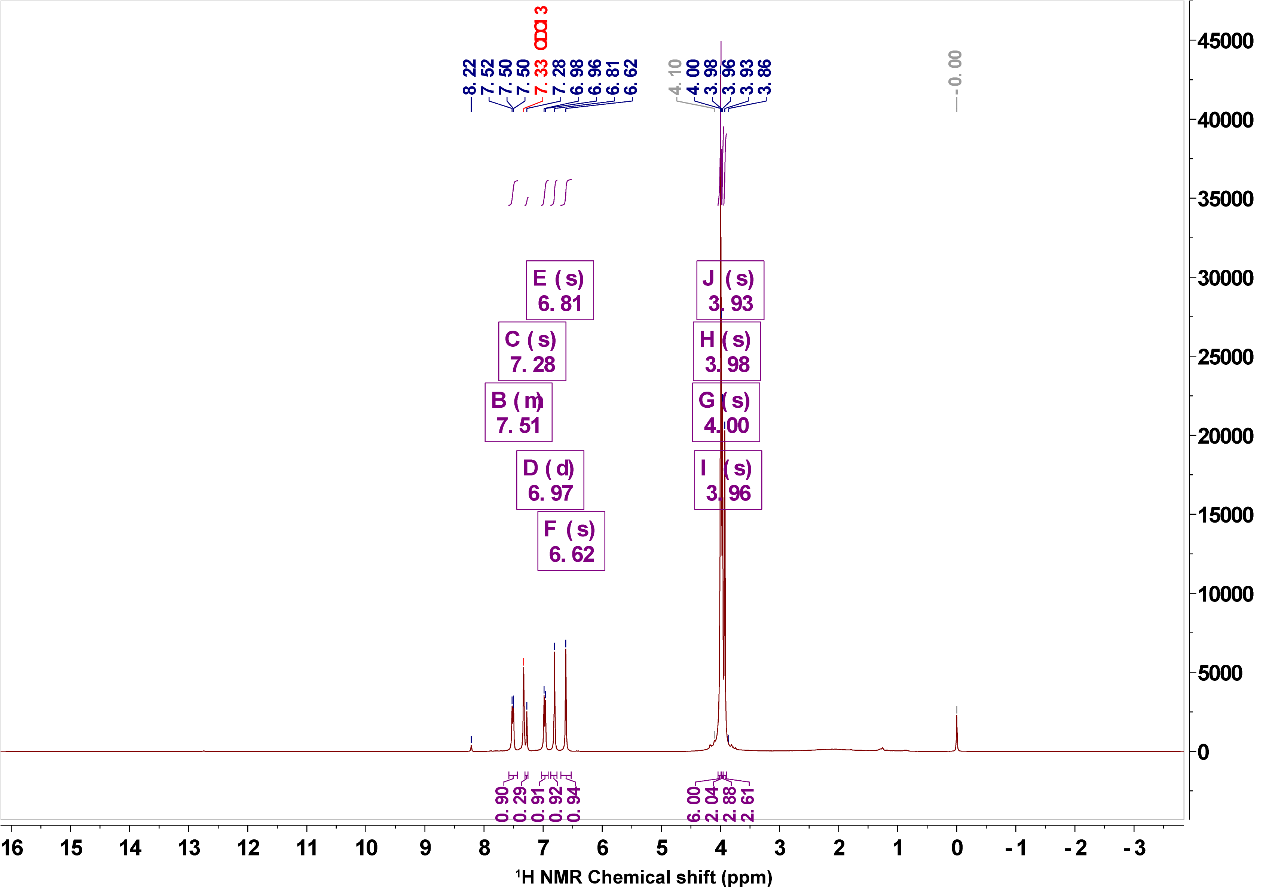


**Figure S2.** ^1^H-NMR spectrum of sinensetin


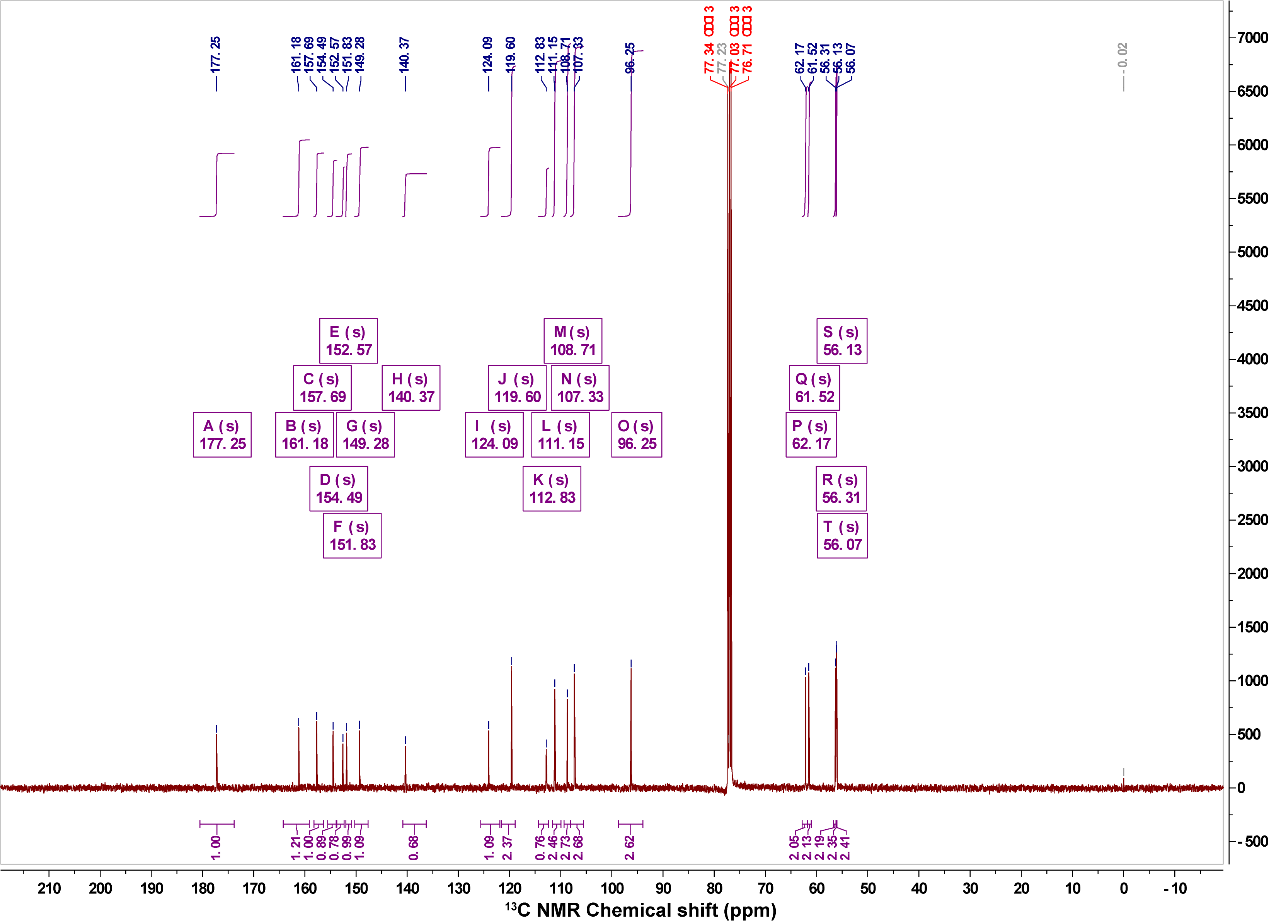


**Figure S3.** ^13^C-NMR spectrum of sinensetin


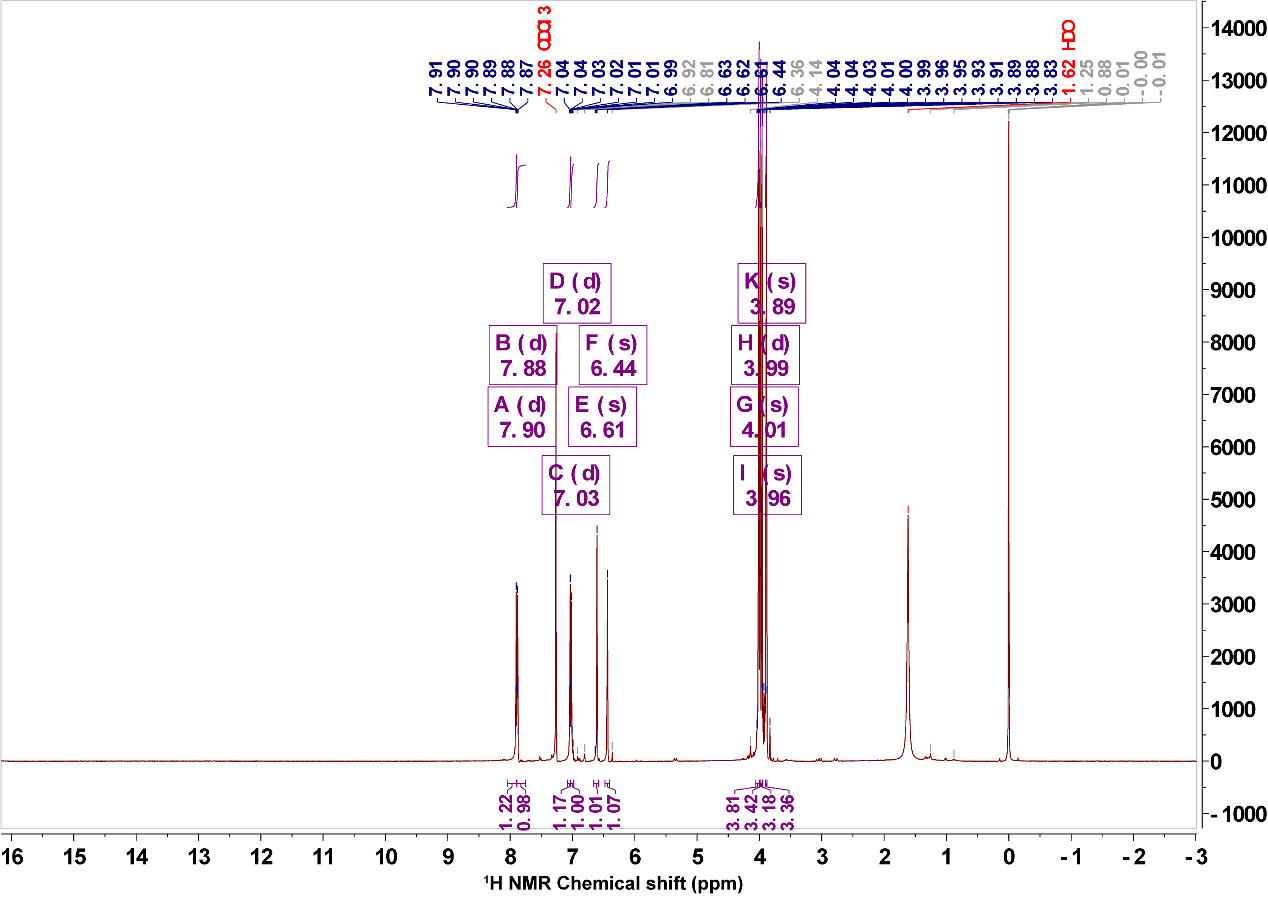


**Figure S4.** ^1^H-NMR spectrum of 5,6,7,4′-tetramethoxyflavone


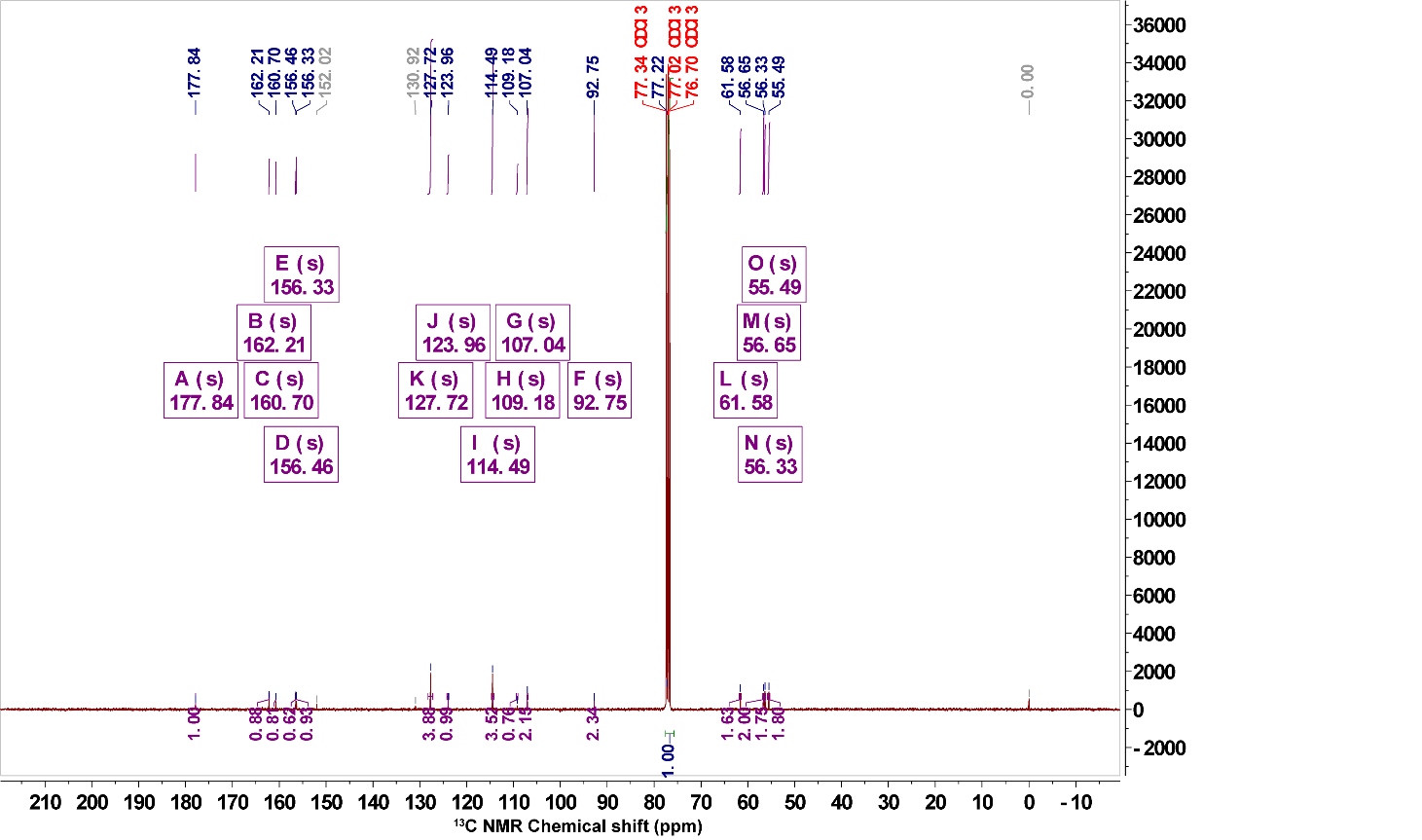


**Figure S5.** ^13^C-NMR spectrum of 5,6,7,4′-tetramethoxyflavone


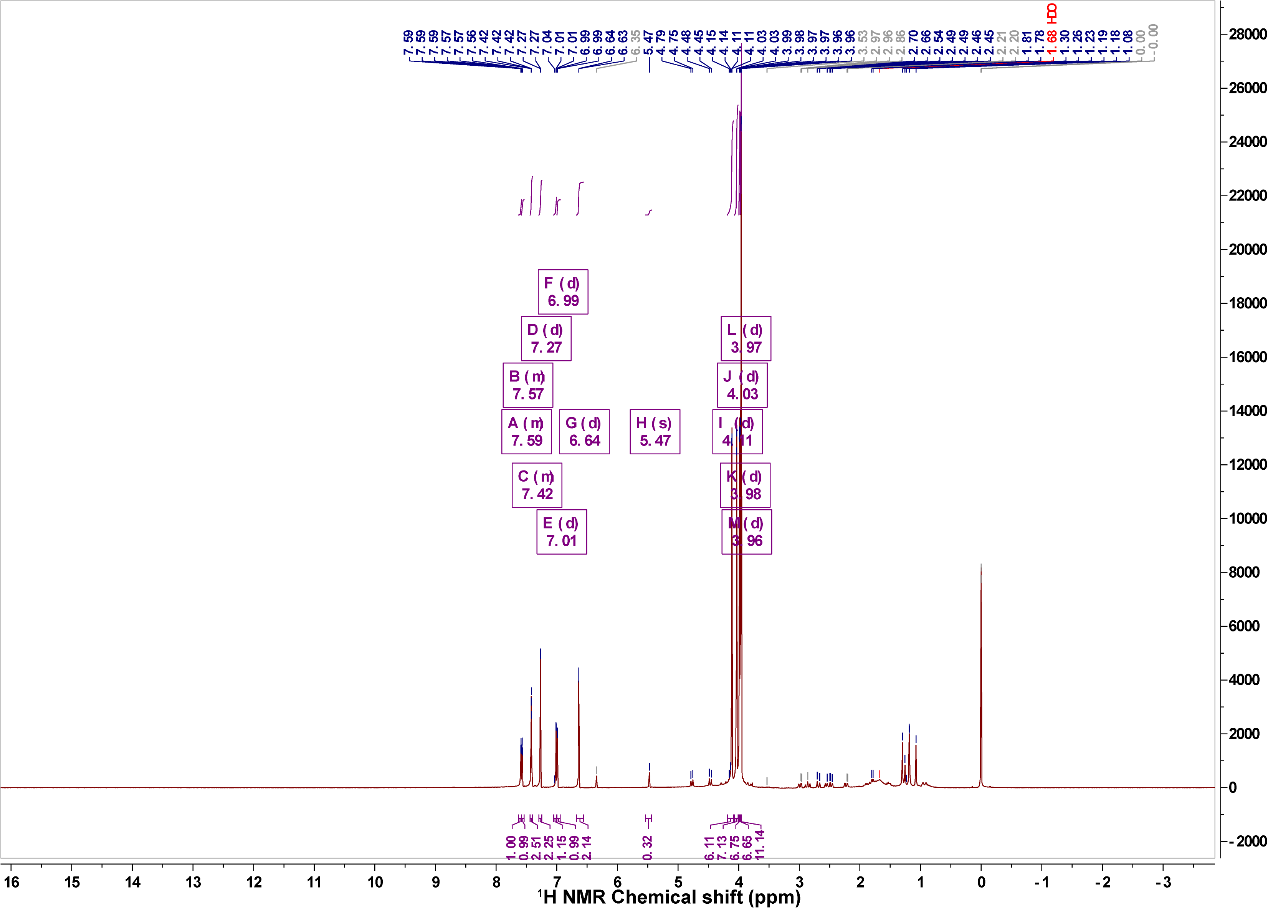


**Figure S6.** ^1^H-NMR spectrum of nobiletin


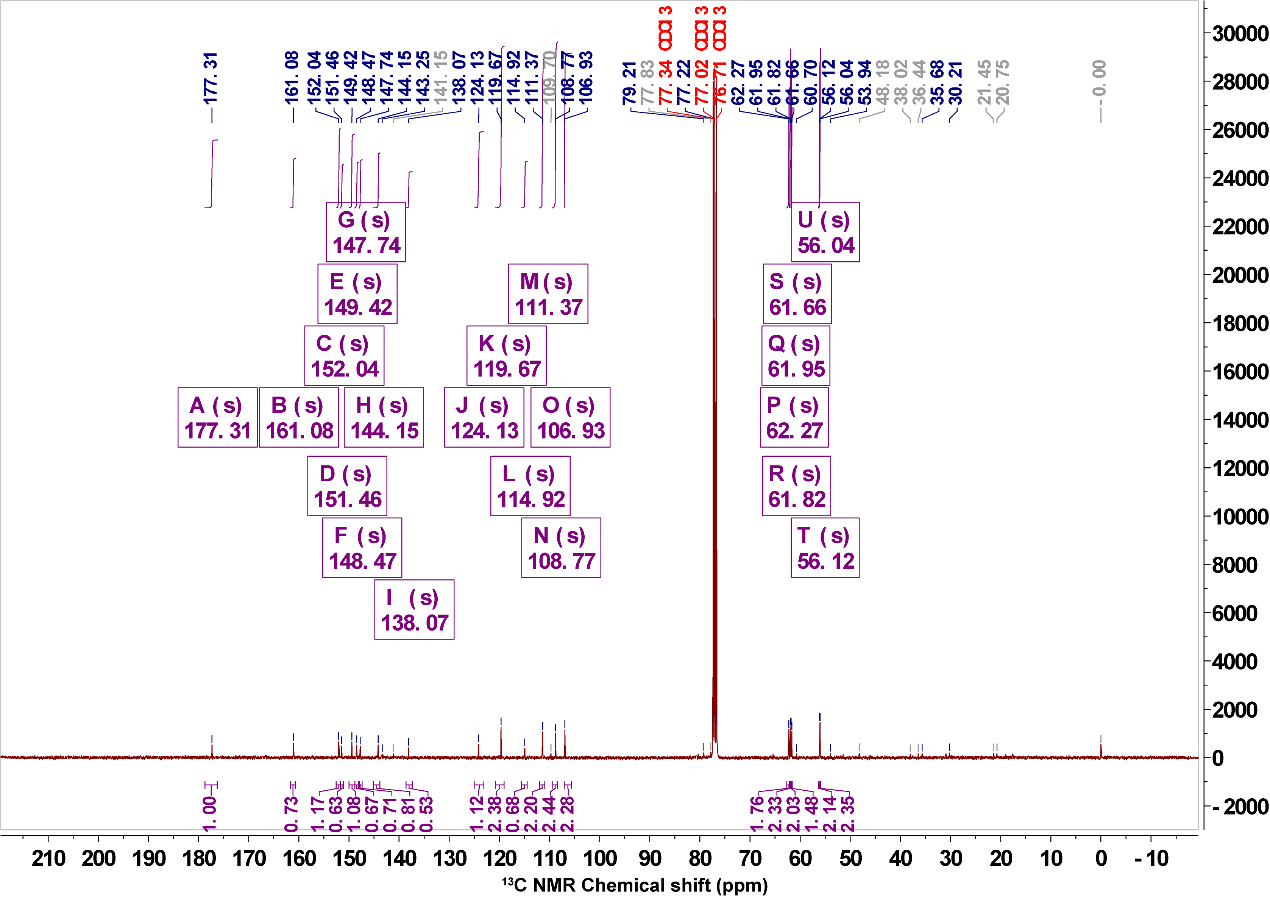


**Figure S7.** ^13^C-NMR spectrum of nobiletin


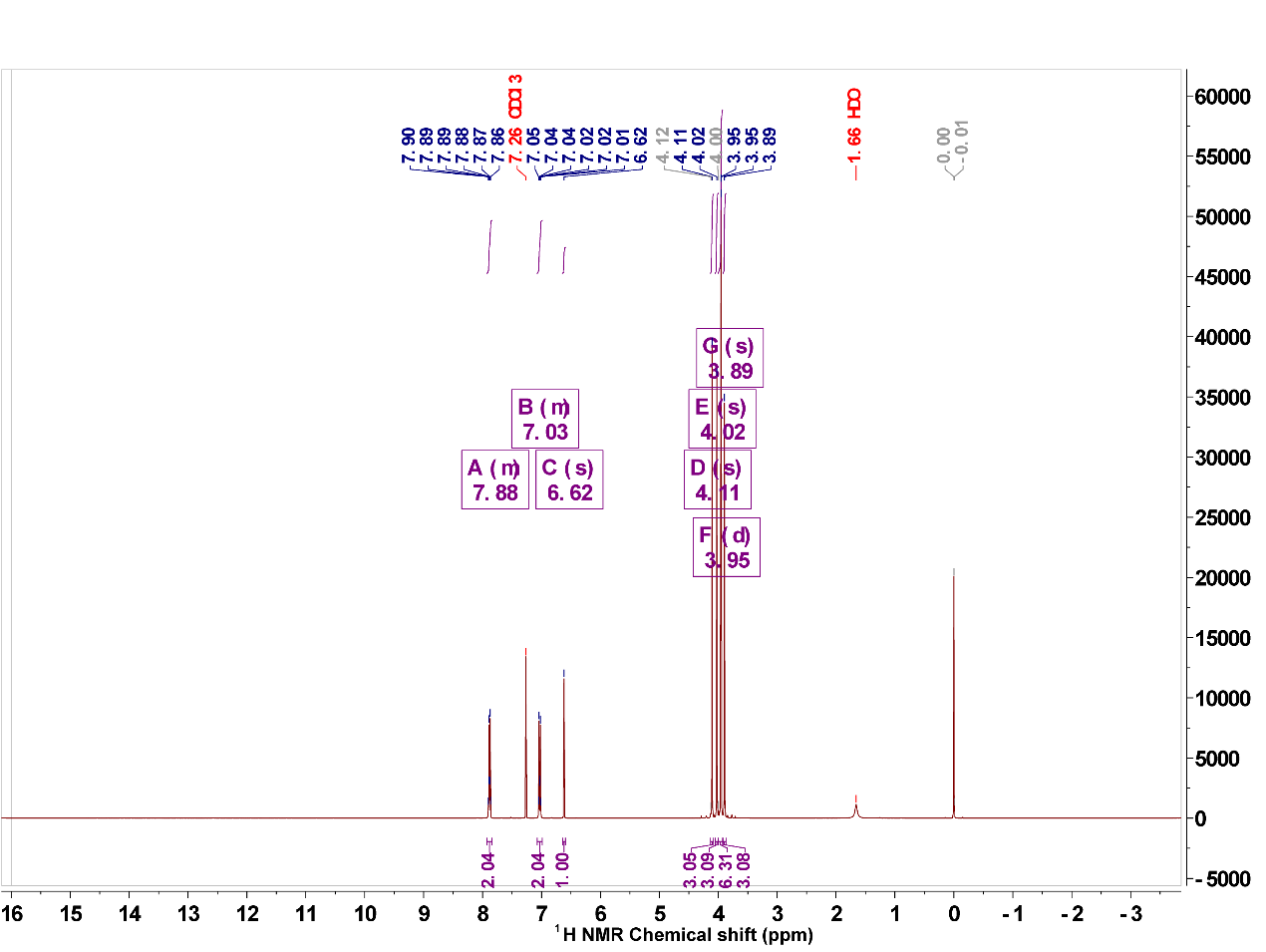


**Figure S8.** ^1^H-NMR spectrum of tangeretin


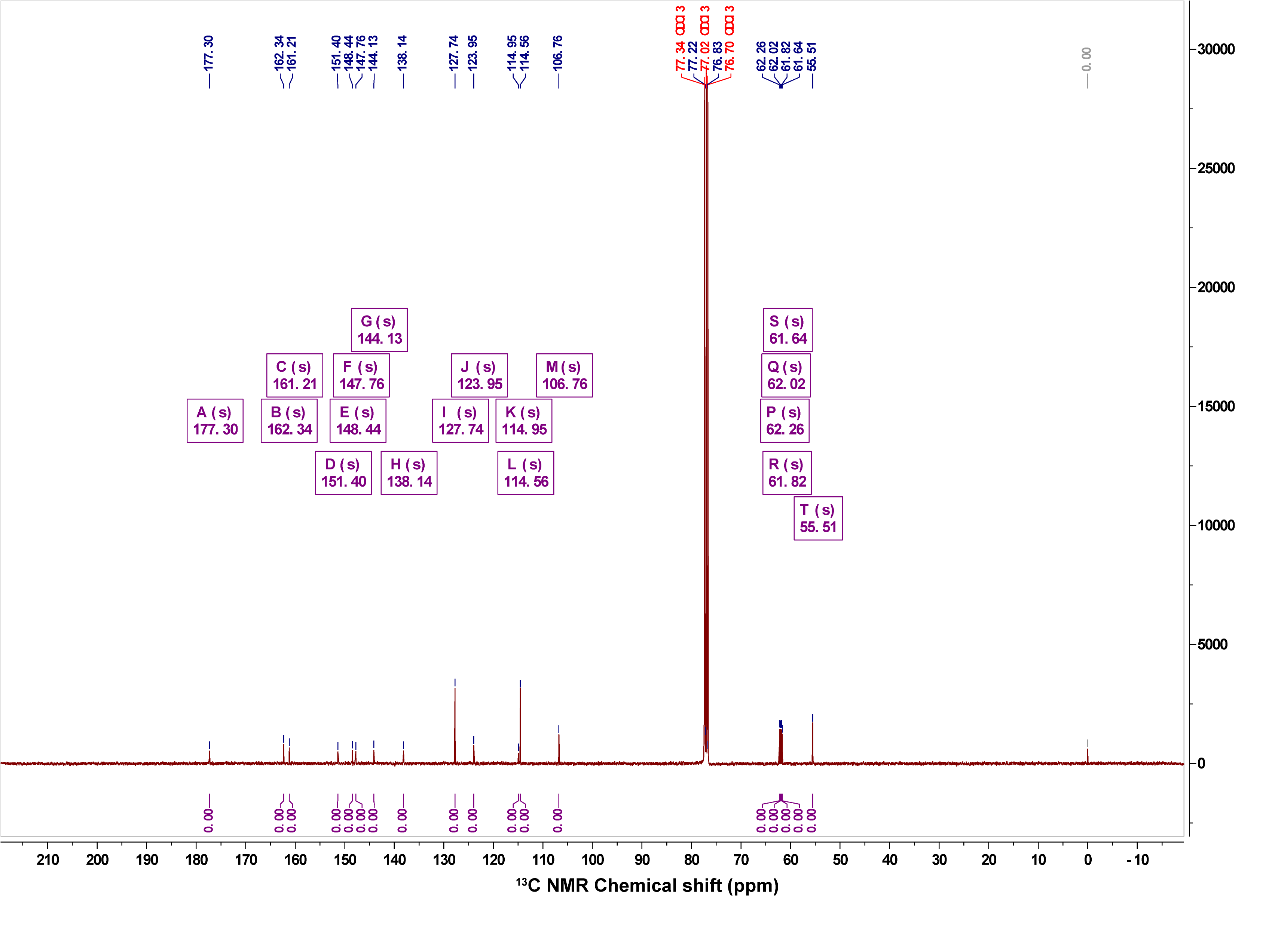


**Figure S9.** ^13^C-NMR spectrum of tangeretin


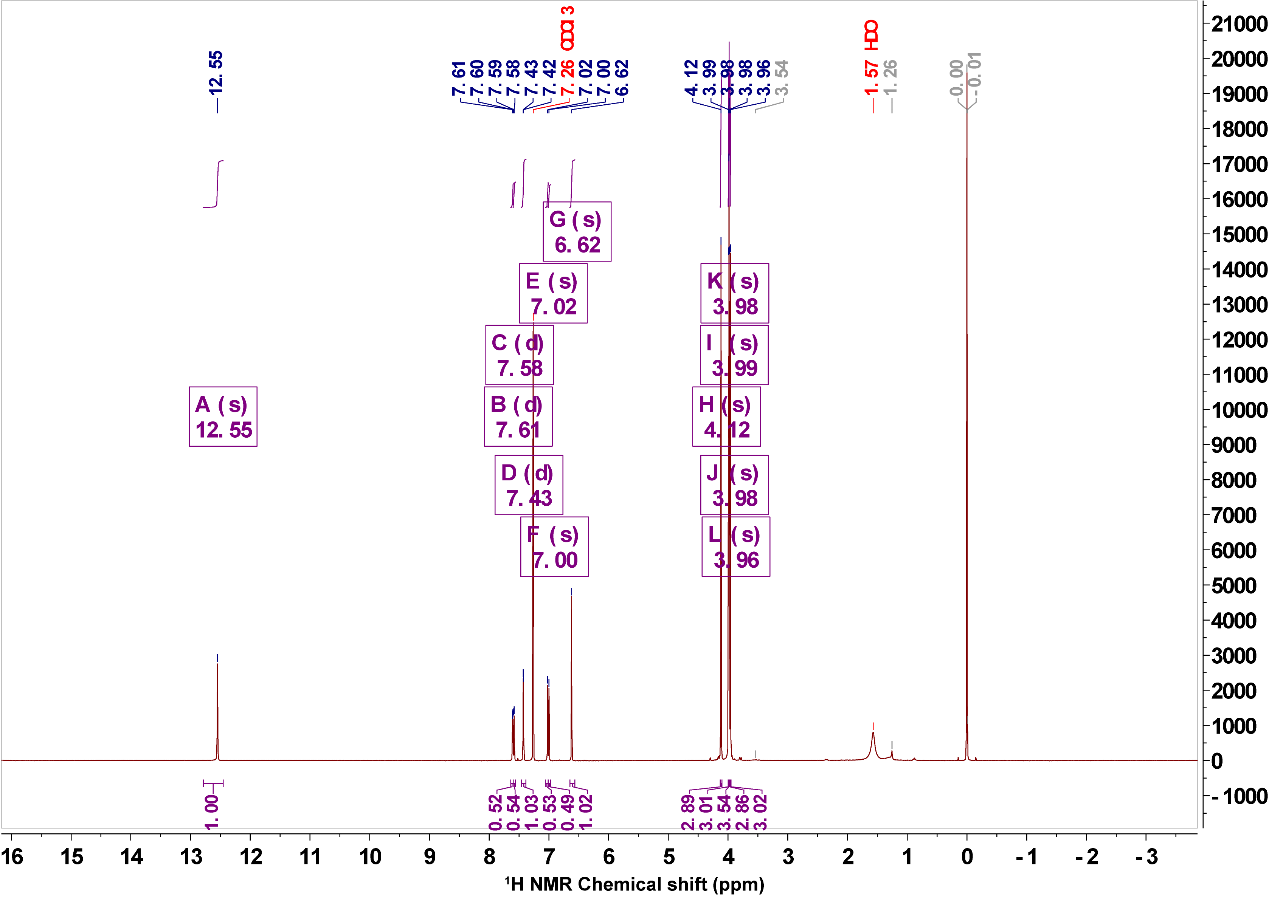


**Figure S10.** ^1^H-NMR spectrum of 5-OH-6,7,8,3′,4′-pentamethoxyflavone


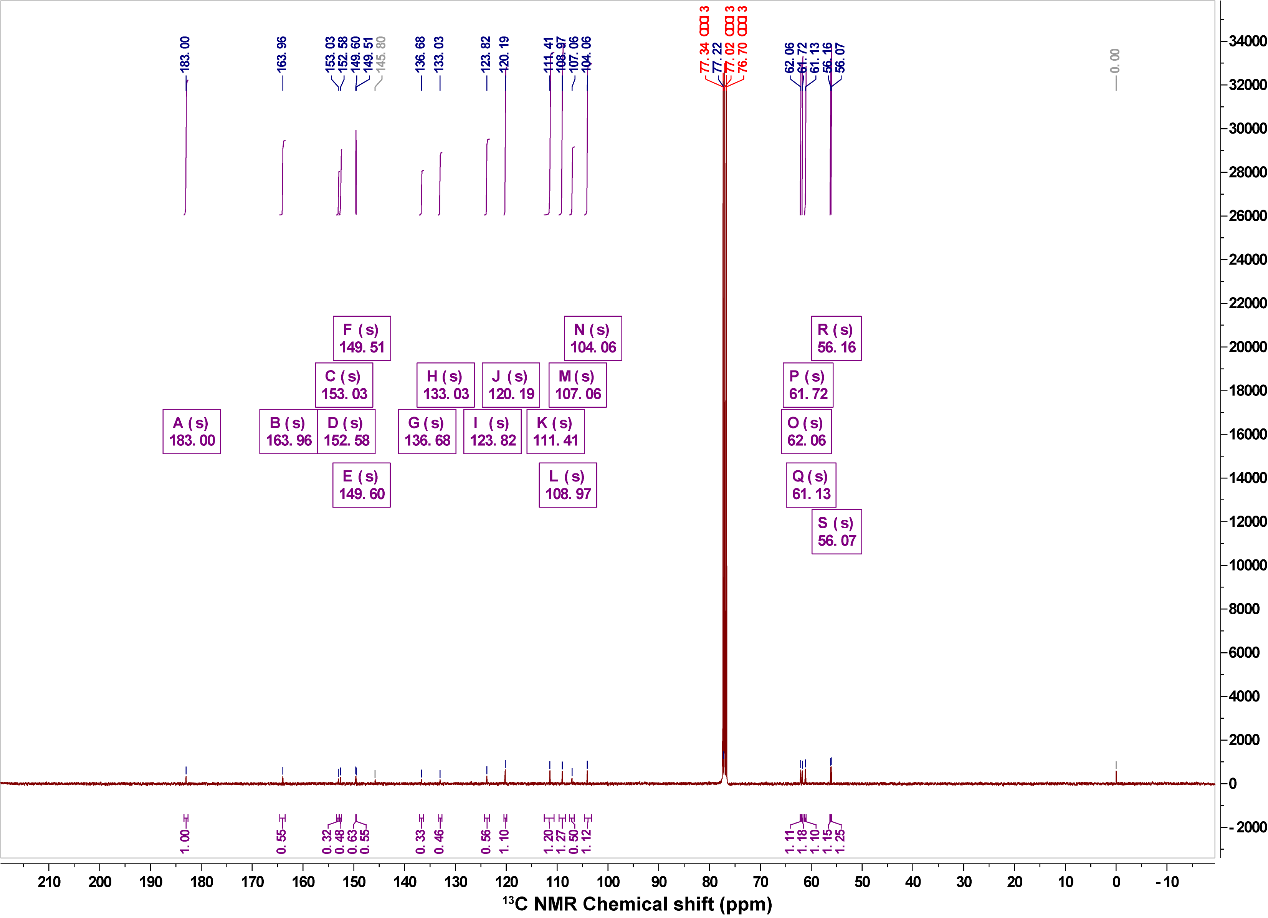


**Figure S11.** ^13^C-NMR spectrum of 5-OH-6,7,8,3′,4′-pentamethoxyflavone

## Supplementary Tables

**Table S1.** Information of quantitative PCR primers

| Genes | Sequence of primer (5'-3') | |
| --- | --- | --- |
| Antiinflammatory-related genes | |  |
| *COX2* | F: TGGTGCCTGGTCTGATGATG | R: GTGGTAACCGCTCAGGTGTTG |
| *iNOS* | F: CCCTCCTGATCTTGTGTTGGA | R: TCAACCCGAGCTCCTGGAA |
| *β-actin-1* | F: AACCGTGAAAAGATGACCCAGAT | R: CACAGCCTGGATGGCTACGT |
| Anticancer-related genes | |  |
| *PTEN* | F: GACGGGAAGACAAGTTCAT | R: GGTTTCCTCTGGTCCTGGT |
| *Akt 1* | F: ATGAGCGACGTGGCTATTGT | R: GAGGCCGTCAGCCACAGTCT |
| *Akt 2* | F: AACCTGTGCTCCATGACCTC | R: CCCTTCTACAACCAGGACCA |
| *Bcl-2* | F: ATGTGTGTGGAGAGCGTCAA | R: ACAGTTCCACAAAGGCATCC |
| *BAX* | F: TTGCTTCAGGGTTTCATCCA | R: CAGCCTTGAGCACCAGTTTG |
| *Caspase-3* | F: GGCATTGAGACAGACAGTGG | R: CATGGAATCTGTTTCTTTGC |
| *β-actin-2* | F: TGCCCATCTACGAGGGGTATG | R: CTCCTTAATGTCACGCACGATTTC |

**Table S2.** Concentrations of Individual PMFs in the peel extract of *Citrus tangerina* ‘Dahongpao’

| **No.** | **RT (min)** |  | **Compound** | **Content (mg/g DW)** |
| --- | --- | --- | --- | --- |
| 1 | 5.33 |  | isosinensetin | 12.90 ± 0.12 |
| 2 | 5.48 |  | sinensetin | 28.11 ± 1.11 |
| 3 | 5.79 |  | 5,6,7,4′- tetrathoxyflavone | 12.14 ± 0.36 |
| 4 | 5.92 |  | nobiletin | 210.87 ± 0.57 |
| 5 | 6.06 |  | 3,5,6,7,8,3′,4′- heptamethoxyflavone | 7.68 ± 0.46 |
| 6 | 6.29 |  | 5-hydroxy-6,7,8,3′,4′-pentamethoxyflavone | 24.66 ± 0.33 |
| 7 | 6.34 |  | tangeretin | 55.66 ± 0.62 |

**Table S3.** IC_50_ (µM) for PC-3 and DU145 cell lines of individual citrus PMFs

| PMFs | DU145 | PC-3 |
| --- | --- | --- |
| tetramethyl-O-scutellarin | - | - |
| 5-demethylnobiletin | 54.14 | 49.33 |
| Tangeretin | 46.60 | 22.12 |
| Nobiletin | - | - |
| Sinensetin | - | - |

Note: "-" means that the inhibition of PC-3 and DU145 cells has not reached half.
